# Complications of percutaneous vertebroplasty: An analysis of 1100 procedures performed in 616 patients

**DOI:** 10.1097/MD.0000000000003850

**Published:** 2016-06-17

**Authors:** Agnieszka Saracen, Zbigniew Kotwica

**Affiliations:** Faculty of Health Sciences and Physical Education, Kazimierz Pulaski University of Technology and Humanities, Radom, Poland.

**Keywords:** cement leakage, neoplastic fractures, osteoporotic fractures, percutaneous vertebroplasty, traumatic compression fractures, VAS scale, vertebral hemangioma

## Abstract

Percutaneous vertebroplasty (PVP) is a minimally invasive procedure widely used for the treatment of pain due to vertebral fractures of different origins—osteoporotic, traumatic, or neoplastic. PVP is minimally invasive, but the complications are not rare; however, they are in most cases not significant clinically. The most frequent is cement leakage, which can occur onto veins, paravertebral soft tissue, into the intervertebral disk, or to the spinal canal, affecting foraminal area or epidural space. We analyzed results of treatment and complications of vertebroplasty performed with the use of polimethylomethylacrylate cement (PMMA) on 1100 vertebrae, with a special regard to the severity of complication and eventual clinical manifestation. One thousand one hundred PVP were analyzed, performed in 616 patients. There were 468 (76%) women and 148 men (24%), 24 to 94-year old, mean age 68 years. From 1100 procedures, 794 treated osteporotic and 137 fractures due to malignant disease, 69 PVP were made in traumatic fractures. One hundred patients had painful vertebral hemangiomas. Seven hundred twenty-six (66%) lesions were in thoracic, and 374 (34%) in lumbar area. Results of treatment were assessed using 10 cm Visual Analogue Scale (VAS) 12 hours after surgery, 7 days, 30 days, and then each 6 months, up to 3 years. Before surgery all patients had significant pain 7 to 10 in VAS scale, mean 8.9 cm. Twelve  hours after surgery 602 (97.7%) reported significant relief of pain, with mean VAS of 2,3 cm. Local complications occurred in 50% of osteoporotic, 34% of neoplastic, 16% of traumatic fractures, and 2% of vertebral hemangiomas. The most common was PMMA leakage into surrounding tissues—20%; paravertebral vein embolism—13%; intradiscal leakage—8%; and PMMA leakage into the spinal canal—0.8%. Results of treatment did not differ between patients with and without any complications. From 104 patients who had chest X-ray or CT study performed after surgery, pulmonary embolism was noted in 2 patients, but without any clinical symptoms. Only 1 patient—with PMMA leakage into the spinal canal required surgical decompression In conclusion, PVP is effective in decreasing the level of pain in compression vertebral fractures. Complications occur in almost half of the patients but in more than 95% of them do not produce any clinical symptoms.

## 1 Introduction

With the aging population, the incidence of osteoporotic or neoplastic vertebral compression fractures (VCF) is increasing and becoming a major health-care issue.^[1]^ Percutaneous vertebroplasty (PVP) is a minimally invasive procedure widely used for the treatment of pain due to vertebral fractures of different origin—osteoporotic, traumatic, or neoplastic.^[2–7]^ It is also a method of choice in the treatment of painful vertebral hemangiomas.^[8]^ PVP is minimally invasive, but the complications are not so rare; however, they are, in most cases, not significant clinically.^[2,3,8–10]^ The most frequent is cement leakage,^[9,11,12]^ which can occur onto veins,^[13]^ paravertebral soft tissue, into the intervertebral disk, or to the spinal canal, affecting foraminal area or epidural space.^[9,14–17]^ Extremely rare intradural leakage can occur.^[18,19]^ PVP can also increase the risk of fractures of adjacent vertebrae.^[14,20–23]^ The incidence of postoperative infections is low.^[24]^ Systemic complications are rare,^[25,26]^ but can produce serious diseases—fat embolism,^[27]^ PMMA pulmonary embolism,^[25,28–30]^ cardiac damage,^[31,32]^ or arterial^[33]^ or renal embolization.^[34]^ Epidural hemorrhage can occur, but is extremely rare.^[35]^

There are hundreds reports on the beneficial effect pf PVP; however, there are also reports putting in doubt the efficacy of vertebroplasty in VCF. Some articles revealed no efficacy of PVP in comparison with sham-operated patients.^[36–38]^ Later studies of the same authors showed no or limited value of vertebroplasty in long-term follow-up studies.^[38–40]^

In this study, we analyzed results of treatment and complications of vertebroplasty performed with the use of polimethylomethylacrylate cement (PMMA) on 1100 vertebrae, with a special regard to the clinical manifestation and severity of complications of PVP.

## 2 Methods

One thousand one-hundred PVP were analyzed, performed in 616 patients. The patients were treated between 2006 and 2012 in 2 neurosurgical departments, but were operated by the same neurosurgeons. All treated patients were included into the study, the only 1 criteria was the presence of vertebral fracture and treatment by PVP. There were 468 (76%) women and 148 men (24%), 24 to 94-year old, mean age 68 years. Three hundred sixty patients had osteoporotic fractures, 200 single, and 160—2 to 6 fractures. Ninety patients had malignant disease, 32 myeloma, 36 prostate carcinoma, 20 breast, and 2 pulmonary cancer metastases. One hundred patients had painful vertebral hemangiomas, and 66 patients painful posttraumatic fractures. From 616 patients with painful fractures, 417 patients had 1, and 199 more than 1 fracture, up to 6. Age, gender, the cause, and number of fractures are shown in Tables 1 and 2.

**Table 1:**
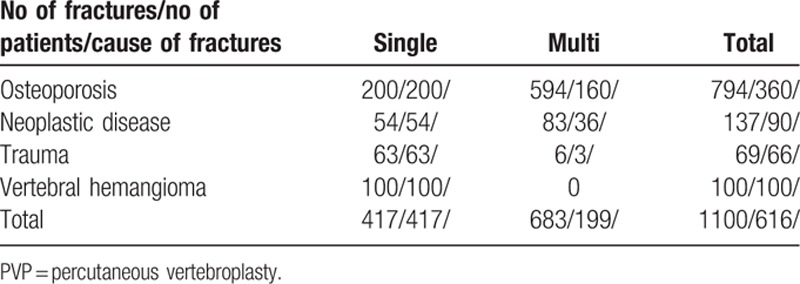
The cause of PVP and number of affected vertebrae.

**Table 2:**
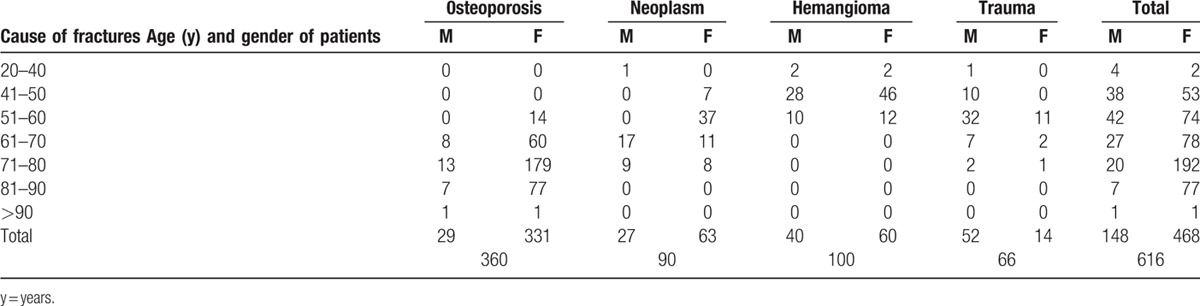
Age and gender of patients according to the cause of vertebral fractures.

The study was approved by the Ethics Committee of Faculty of Health Sciences, University of Technology and Humanities, Radom/n^o^ WNZKF12/01/.

All the patients were admitted to the hospital because of severe pain or intense aggravation of chronic pain. Patients were admitted with CT or MR performed in last 2 weeks before hospitalization. All single fractures were diagnosed as acute, with time from the beginning of pain up to 6 weeks. In multiple fractures, according to the clinical examination and radiological picture 1 level was defined as fresh fracture, all the others were assigned as chronic. In 126 from 160 patients with multiple fractures we found the history of diagnosed fractures up to 2 years. All these 126 patients were previously treated conservatively and all of them complained of chronic pain which aggravated within some weeks before PVP. In all 616 patients an intensive pain developed from 2 to 6 weeks before augmentation.

From 1100 procedures, 794 treated osteporotic, 137 fractures due to malignant disease. Sixty-nine PVP were made in traumatic fractures. One hundred patients had solitary large, painful vertebral hemangiomas. Seven hundred twenty-six (66%) lesions were in thoracic, and 374 (34%) in lumbar area. All fractures were compression ones.

All procedures were performed under local anesthesia with lidocaine. The patients were in prone position and vertebroplasty was performed with a 11- or 13-gauge needle, under biplane fluoroscopy. In all patients unilateral transpedicular approach was used. Volume of PMMA injected was 0.5 to 3 mL in thoracic, and 0.5 to 3 mL in lumbar area. The volume of PMMA was low, in the first years we used up to 3 mL of PMMA, since 2009 the volume has not exceeded 2 mL in lumbar and 1.5 mL in thoracic area. If the height of the vertebra was very low—vertebra plana, PMMA volume usually did not exceed 0.5 mL was injected with the use of normal 10 mL syringe, because we believe it allows to control precisely the pressure of injection. We never used liquid cement, we always injected cement of medium viscosity. In patients with multiple fractures the decision was to augment all fractured vertebrae, but during 1 session maximally 3 vertebrae were augmented, and eventual next surgical treatment was postponed for 2 to 4 weeks. During the first procedure PVP was always performed at the level clinically producing the most intensive pain.

Results of treatment were assessed using 10 cm Visual Analogue Scale (VAS) 12 hours after surgery, 7 days, 30 days, and then each 6 months, up to 3 years. We only assessed the level of pain located in vertebral column area. Patient's functioning and their quality of life were not taken into account in this paper, because prevailing number of the patients were old, and their functioning as well as quality of life depended on many other factors, including medical and social ones.

Comparisons between groups were performed with the use of *t* test and *P* values of <0.01 were deemed significant.

## 3 Results

Before surgery all patients had significant pain 7 to 10 in VAS scale, mean 8.9 cm. Twelve  hours after surgery 602 (97.7%) reported significant relief of pain, with mean VAS of 2.3 cm. Fourteen patients had no relief, 8 of them had intracanal leakage, the other 4 had leakage into surrounding tissues, and the last 2 had no complications. 30th day examination revealed that from 8 patients with leakage onto the spinal canal, 6 presented significant relief of pain, in the last 2 the pain persisted at the same level. Three years follow-up was achieved in 512 patients, we lost contact with 104 patients (17%). More than 90% reported relief of pain, and VAS after 3 years slightly changed, rising from 2.3 cm directly after surgery to 3.2 cm after 3 years. New fractures were found in 64 patients, in 11 in vertebrae adjacent to previously augmented. New fractures occurred in 20 of 32 patients with myeloma, and 44 osteoporotic patients. In all patients with new osteoporotic fractures the next procedures were performed and in all the result of treatment was satisfactory. Results of treatment according to VAS values are summarized in Table 3.

**Table 3:**
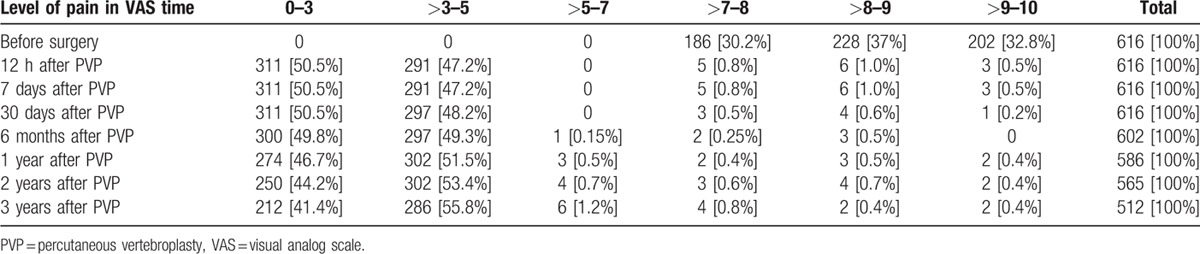
Results of treatment in 3-year follow-up according to visual analog scale.

Different complications were noted in 459 augmented vertebrae—41.7% of all procedures. Leakage occurred in 50% of osteoporotic fractures, in 34% of neoplastic, and in 16% of traumatic fractures. In vertebral hemangiomas we observed paravertebral vein embolism in 2 from 100 patients, without any other complications. In neoplastic and osteoporotic patients surrounding tissue leakage and paravertebral vein embolism were the most common PVP complications. In 5% to 10% of patients from all groups except patients with hemangiomas, intradiscal leakage was seen, without any clinical symptoms. From 96 patients with osteoporotic fractures with symptoms of Kummel disease, in 23 (24%) intradiscal leakage was observed, with no other complications.

In 9 patients intracanal leakage occurred, in 7 with neoplastic, and in 2 with osteoporotic fractures. One patient, with multiple myeloma, developed monoparesis of the lower extremity and required decompressive laminectomy. The other 8 patients did not benefit from the procedure in a short time after procedure, but 30th day examination revealed that 6 of them presented significant relief of pain, only 2 reported no pain relief.

One hundred four patients had pulmonary X-ray or CT made during the time of observation. In 2 patients pulmonary embolism was observed, in 1 with osteoporotic fracture and with no symptoms of extravertebral leakage, and in 1 patient with myeloma, who had leakage into the spinal canal. In both patients the area of embolism was minimal, and did not exceed several millimeters. They also did not present any clinical signs of pulmonary embolism. Leakage into spinal canal was significantly more often in neoplastic diseases (*P* <0.01), leakage into the surrounding tissues was significantly most often in osteoporotic fractures, embolization of paravertebral veins was more often in osteoporosis and neoplastic diseases, than in other reasons of vertebroplasty (*P* <0.01). Significantly more often (*P* <0.01) intradiscal leakage was observed in patients with Kummel disease. The complication rate was highest in osteoporosis, and then in neoplastic disease. Vertebroplasty practically did not show any complications in patients with painful hemangiomas. Incidence of complications was the same in thoracic and lumbar area, there were no statistical differences between these locations. Analysis of the age and number of complications did not reveal any differences between different ages and sex groups. There were no statistical differences in treatment results between patients with and without complications of augmentation. Table 4 summarizes all the complications noted.

**Table 4:**
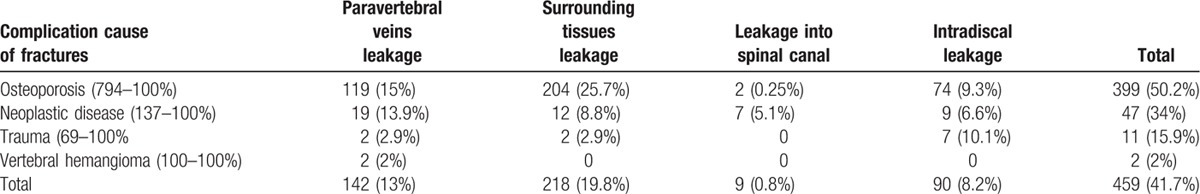
Complications of vertebroplasty in 1100 augmented vertebrae.

## 4 Discussion

PVP is relatively safe, but it can be associated with severe clinical complications—spinal cord compression^[15–19]^ or systemic embolism^[13,30,31,33,34]^; or less severe—infection,^[24]^ or radicular pain.^[41]^ Local leakage of PMMA is frequent, up to 90% of patients,^[10]^ but in most cases does not produce any symptoms.^[2–3,5]^ Leakage is more frequent in malignant lesions treatment, because of possible destruction of the cortex of vertebral body and higher vascularization.^[9,12]^

Despite some controversy concerning the efficacy of vertebroplasty,^[1,5,36–40]^ most reports point out its beneficial effect on pain.^[3,5,7,22,23]^ Significant pain reduction appears within hours after surgery and lasts for a long time.^[3,5,7,8]^ It also gives relief of pain in long follow-up studies. Compression fractures can also be treated with other methods. Open surgery and stabilization brings a significant number of complications, especially in osteoporosis and neoplastic disease.^[3,5]^ Conservative treatment, including pain killers and external stabilization, gives relief after some weeks, and the complication can be severe, including pulmonary embolization, pulmonary infections, due to immobilization by pain.^[1,3]^ The number of gastric complications caused by extensive doses of pain killers is also significant.^[23]^ In malignant disease radiation therapy is effective; however, the relief of pain is postponed for some weeks.^[9,12]^ Kyphoplasty gives similar results to vertebroplasty, but is much more expensive. The advantage of PVP and kyphoplasty over other methods of treatment is a very effective pain reduction appearing in a very short time after surgery what can minimize eventual complications due to immobilization by pain.^[23]^

Vertebroplasty is minimally invasive; however, the number of complications, especially extravertebral PMMA leakage, is significant, from several up to more than70%.^[1–3,5,8]^ In our material complications were noted in 42% of all performed procedures. However, 99% did not produce any clinical symptoms. The effect of treatment was beneficial in more than 95% and there was no difference in pain reduction between patients with leakage noted and patients without any complications. In almost all the patients cement leakage was harmless and required no further therapy. The only 1 exception was leakage into the spinal canal. It occurred in 9 of 1100 patients, and in 1 patient produced severe complications—monoparesis and extensive sensory disturbances. In patients with narrowing of intervertebral foramens immediately after surgery no pain reduction was observed, but 1 month later, the patients also reported significant relief of pain. Leakage into the spinal canal took place in 7 of 90 patients with neoplastic fractures and was significantly more often than in patients with other indications for treatment. Leakage into soft tissues and embolization of paravertebral veins occurred in almost 50% procedures; however, no one patient showed any clinical symptoms. Such leakage occurs in osteoporosis significantly more frequent than in other indications for augmentation.

Pulmonary embolism occurs in 4 up to 26% of patients.^[25,28–30]^ In our material no one patient, during the time of follow-up, did reveal any systemic complications. During follow-up 104 patients had X-ray study of lungs performed and in 2 of them small acrylic deposits were found. One of these patients had extradural leakage into the spinal canal. In both patients no clinical symptoms of pulmonary embolization occurred.

Literature data show different complications of PVP, including infections, epidural hemorrhage,^[35]^ fat embolism,^[27]^ cardiac damage,^[31,32]^ or arterial^[33]^ or renal embolism.^[34]^ These complications are extremely rare, and we did not observe them in our practice.

PMMA volume used for the procedure can be limited to low amounts. In the beginning, we used 2 to 3 mL of PMMA for each procedure; however, since 2009 we have limited PMMA volume to maximally 2.0 mL, obtaining similar results as before. It strictly corresponds to observations of Hussain and Erdek^[1]^ that small amounts of PMMA significantly reduce pain but can minimize a number of complications of PVP.

During 3-year follow-up, 64 patients developed new fractures, 11 in vertebrae adjacent to previously augmented. New fractures developed in 1 of 3 of patients with multiple myeloma and in 12% of osteoporotic patients. It does not support the thesis that PVP increases new compression fractures in adjacent vertebrae.^[1,21–23]^

In this study, we present results of PVP in different types of vertebral compression fractures. All the patients were treated surgically and thus we cannot compare them to any control groups—sham-operated or treated conservatively patients. Some studies stated no benefit of PVP in comparison to sham procedures; in the other study PVP prevailed as compared with sham-operated patients.

It must be pointed out that significant relief of pain appears within hours after PVP, while in conservatively treated patients or in sham-operated ones the relief of pain is postponed for some to several weeks.^[36–40]^ Despite lack of control group, what we understand can be seen as important limitation of the study we think that our conclusion about beneficial effect of PVP can be drawn up from our results. It must be also pointed out that studies that put in doubts the effectiveness of PVP based on a very limited group of patients—up to 40 patients in each analyzed group.^[36–38]^

## 5 Conclusions

Verterbroplasty is a minimally invasive surgical procedure, but local complications occur in almost 50% of patients. However, in more than 95% of these complications—mainly leakage into surrounding tissue and embolization of paravertebral veins have no effect on results of treatment—vertebroplasty significantly reduces pain in more than 95% of patients. In 9 (1.5%) patients we found leakage into the spinal canal, but in 1 only clinical symptoms were symptomatic and only this patient required surgical decompression of the spine. It must be pointed out that intracanal leakage occurred almost in fractures caused by osteoblastic tumors only.
